# Editorial: The Evolving Role of Immunotherapy in Non-Melanoma Skin Cancers

**DOI:** 10.3389/fonc.2022.870509

**Published:** 2022-05-18

**Authors:** Michele Guida, Pietro Quaglino, Paola Queirolo

**Affiliations:** ^1^Rare Tumors and Melanoma Unit, Istituto di Ricovero e Cura a Carattere Scientifico (IRCCS) Istituto Tumori Giovanni Paolo II, Bari, Italy; ^2^Dermatologic Clinic, University of Turin Medical School, University of Turin, Turin, Italy; ^3^Melanoma, Sarcoma and Rare Tumors Oncology Department, European Institute of Oncology (IEO), Istituto di Ricovero e Cura a Carattere Scientifico (IRCCS), Milan, Italy

**Keywords:** non melanoma skin cancer, immunotherapy, PD-1, immune suppression, prognosis

Non Melanoma Skin Cancers (NMSCs) represent the most common form of cancer in Caucasians, whose incidence continues to increase all over the world (1, 2). Traditionally, NMSC referred mainly to skin tumours deriving from keratinocytes, thus including basal cell carcinoma (BCC) and cutaneous squamous cell carcinoma (cSCC), that represent the most frequent subtypes. In addition to BCC and cSCC, this group also includes Merkel cell carcinoma (MCC), dermatofibrosarcoma protuberans, adnexal carcinoma, atypical fibroxanthoma, soft tissue sarcomas including angiosarcoma and, in particular, Kaposi sarcoma, and primary cutaneous lymphoma.

From an epidemiological point of view, BCC represents the most frequent malignant tumour type in humans, followed by cSCC. On the other hand, other NMSCs such as Merkel cell carcinoma or primary cutaneous lymphoma are very rare although their incidence is rapidly increasing. NMSCs mainly affects elderly people, and the most frequent cutaneous sites of development are the head and neck area (BCC, cSCC and MCC). Immunosuppression is an important risk factor for developing these tumours.

Significant differences can be found between these tumour types in terms of disease course and survival. More than 90% of patients with cSCC are disease-free after surgery at a 5-year follow-up, however, a percentage of patients ranging from 1.9% to 4.6% develop disease recurrence or progression (3–5). BCC is more frequent overall than cSCC, and it is characterised by a very indolent disease course, with only 1% of cases progress to advanced disease (6). MCC is characterised by a highly aggressive disease course, as more than half of patients show metastatic disease at the initial diagnosis (7, 8). Survival of metastatic patients in the era pre-check point inhibitors was poor, with 18% at 5 years for distant metastatic disease (9, 10).

Surgery represents the treatment of choice, often associated with radiotherapy. However, in case of locally advanced or metastatic forms (1% of BCCs, in 5% of cSCCs and up to 50% of MCCs) these traditional therapeutic approaches are not sufficient for complete disease control.

In case of advanced disease, systemic therapy is a possible choice. With the advent of immune-checkpoint inhibitors (ICIs), previously unexplored horizons have opened up for these pathologies.

Although the majority of NMSC are treated with conventional surgery and/or radiotherapy, a small percentage of patients progress to locally advanced or metastatic disease, mainly due to patient negligence, comorbidities, or immunosuppression. In these circumstances, systemic treatment may be indicated. Until recently, effective therapy remained an area of significant unmet clinical need. Improved understanding of molecular and immune pathogenesis has been critical to driving and developing new therapeutic advances, particularly towards immunotherapy.

The rationale for the application of immunotherapy in NMSC is based on three group of factors: molecular, pathologic and clinical (11–13).

Tumour mutational burden (TMB) measures the quantity of somatic mutations found in a tumour and has been attributed to both endogenous factors and environmental damage. A number of clinical trials have revealed that TMB is correlated with the rate of response to anti-PD-1/PD-L1 blockade (11). Both BCC and cSCC show a marked UV-signature, thus it is conceivable that these cancers exhibit the highest TMB among all other cancer types. The increased expression of neo-antigens which is associated with a high TMB, which likely results in higher levels of tumour neo-antigens that may be targets for the immune system.

As a second point, from a clinical perspective, the mentioned high incidence of NMSC, in particular cSCC and MCC, with conditions of immune-suppression as well as the poor disease course of these cases highlights the relevance of the host immune response in the development and evolution of these diseases. Even if immune environment plays a major role in both BCC and cSCC, probably cSCC presents a higher immunogenicity than BCC in spite of its higher TMB. This theory could also explain the higher incidence of cSCC in immune-suppressed and transplant patients. As a third point, from a pathologic point of view, these tumours are characterised by a significant expression of the PD-1/PDL-1 axis both in tumour cells and microenvironment in the immune infiltrate (12). Moreover, PD-L1 levels had prognostic clinical relevance in as much as patients with a tumour microenvironment type characterised by high expression of both PD-L1 and TILs had the longest survival. Also concerning cutaneous T-cell lymphoma, it has been shown that specific subtypes of these diseases express PD-1 at high levels (14).

All NMSCs are theoretically suitable for immunotherapy; albeit the robustness of their immunological response is quite different. Moreover, in the face of a powerful immune reaction, many patients progress or do not respond to modern immunotherapy due to resistance or immunoescape mechanisms. To date, Cemiplimab for cSCCs (3) and Avelumab (15) for MCCs have been approved by European Medicines Agency (EMA); recently Food and Drug Administration (FDA) approved Cemiplimab for locally advanced Basal cell carcinoma (LaBCC) (16) and other drugs are studied through several clinical trials.

The aim of this Research Topic is to provide clinicians an overview of innovative systemic treatments for NMSC, mainly oriented towards immunotherapy. Adjuvant and neoadjuvant settings, as well as future therapeutic directions, will also be highlighted.

## Author Contributions

PiQ wrote the text, MG and PaQ contributed to drafting and revising the article. All the authors gave final approval of the version to be published, agreed to the submitted journal, and agree to be accountable for all aspects of the work.

## Conflict of Interest

The authors declare that the research was conducted in the absence of any commercial or financial relationships that could be construed as a potential conflict of interest.

## Publisher’s Note

All claims expressed in this article are solely those of the authors and do not necessarily represent those of their affiliated organizations, or those of the publisher, the editors and the reviewers. Any product that may be evaluated in this article, or claim that may be made by its manufacturer, is not guaranteed or endorsed by the publisher.
